# Tumor-Infiltrating γδ T Lymphocytes: Pathogenic Role, Clinical Significance, and Differential Programing in the Tumor Microenvironment

**DOI:** 10.3389/fimmu.2014.00607

**Published:** 2014-11-24

**Authors:** Elena Lo Presti, Franceso Dieli, Serena Meraviglia

**Affiliations:** ^1^Dipartimento di Biopatologia e Metodologie Biomediche, University of Palermo, Palermo, Italy; ^2^Central Laboratory of Advanced Diagnosis and Biomedical Research (CLADIBIOR), University of Palermo, Palermo, Italy

**Keywords:** γδ T cells, TIL, IL-17, immunosuppression, tumor microenvironment

## Abstract

There is increasing clinical evidence indicating that the immune system may either promote or inhibit tumor progression. Several studies have demonstrated that tumors undergoing remission are largely infiltrated by T lymphocytes [tumor-infiltrating lymphocytes (TILs)], but on the other hand, several studies have shown that tumors may be infiltrated by TILs endowed with suppressive features, suggesting that TILs are rather associated with tumor progression and unfavorable prognosis. γδ T lymphocytes are an important component of TILs that may contribute to tumor immunosurveillance, as also suggested by promising reports from several small phase-I clinical trials. Typically, γδ T lymphocytes perform effector functions involved in anti-tumor immune responses (cytotoxicity, production of IFN-γ and TNF-α, and dendritic cell maturation), but under appropriate conditions they may divert from the typical Th1-like phenotype and polarize to Th2, Th17, and Treg cells thus acquiring the capability to inhibit anti-tumor immune responses and promote tumor growth. Recent studies have shown a high frequency of γδ T lymphocytes infiltrating different types of cancer, but the nature of this association and the exact mechanisms underlying it remain uncertain and whether or not the presence of tumor-infiltrating γδ T lymphocytes is a definite prognostic factor remains controversial. In this paper, we will review studies of tumor-infiltrating γδ T lymphocytes from patients with different types of cancer, and we will discuss their clinical relevance. Moreover, we will also discuss on the complex interplay between cancer, tumor stroma, and γδ T lymphocytes as a major determinant of the final outcome of the γδ T lymphocyte response. Finally, we propose that targeting γδ T lymphocyte polarization and skewing their phenotype to adapt to the microenvironment might hold great promise for the treatment of cancer.

## γδ T Lymphocytes: Antigen Recognition and Effector Functions

T cells carrying the γδ T cell receptor (TCR) are important effector cells that may play a role in the anti-tumor immune response. γδ Cells are not a homogeneous population of cells with a single physiological role. Instead, ever increasing complexity in both phenotype and function is being ascribed to γδ cell subsets from various tissues and locations, both in mice and humans.

γδ T cells account for 1–5% of CD3^+^ T cells in the peripheral blood, but constitute a major subset in other anatomic sites, such as the intestine or the skin [here, however, only in the murine but not in human skin (1)]. In the blood of most healthy individuals, T cells expressing the Vδ2 gene paired with one particular Vγ9 chain (referred to as Vγ9Vδ2 T cells) account for 50 to >90% of the γδ T cell population. In contrast, intestinal intraepithelial γδ T cells frequently express the Vδ1 gene, which can associate with different Vγ elements (1, 2). Vδ1 γδ T cells recognize the MHC class I-related molecules MICA, MICB, and ULBPs, which are expressed on epithelial cells by heat shock or oxidative stress and are constitutively expressed to variable levels on many epithelial and hematopoietic tumor cells (3, 4). It has been debated whether MICA/MICB and ULBPs are directly recognized by the Vδ1 TCR or, indirectly activate Vδ1 T cells upon binding to the stimulatory natural killer (NK) receptor, NKG2D, which is also expressed by the vast majority of γδ T cells.

Vγ9Vδ2 T cells recognize phosphoantigens (PAgs) without requirement for antigen processing and presentation, and MHC restriction. PAgs are pyrophosphates derived from the microbial non-mevalonate isoprenoid biosynthesis pathway (5, 6). Structurally related pyrophosphates are generated in eukaryotic cells through the mevalonate pathway. Micromolar concentrations of endogenous pyrophosphates are required for Vγ9Vδ2 T cell activation and such concentrations are achieved after cellular stress and transformation (7). Given the cross-reactivity between microbial and self PAgs, there is a great interest in elucidating how TCR signaling can be induced by such small molecules. PAgs can directly activate Vγ9Vδ2 T cells, but such activation is greatly enhanced by monocytes and/or dendritic cells (DCs). Hence, either PAgs are presented as cargo to the reactive γδ TCR or their cellular processing somehow sensitizes cell recognition through the engagement of the Vγ9Vδ2 TCR by stabilizing surface expression of a TCR-binding molecules (8). A candidate molecule involved in intracellular PAg processing is the F1-ATPase, which directly binds the Vγ9Vδ2 TCR and interacts with ApppI, an adenosine derivative of IPP (9). Moreover, it has been recently found that PAg-induced Vγ9Vδ2 T cell activation requires butyrophilin 3A1 (BTN3A1) (10–12). Therefore, production of exogenous PAgs or up-regulation of endogenous PAgs in human cells in response to either infections or tumor transformation provokes Vγ9Vδ2 T cell reactivity, albeit at substantially different sensitivity.

Intracellular levels of PAgs can be manipulated by drugs. Aminobisphosphonates, such as zoledronic acid, which are in clinical use for the treatment of osteoporosis and bone metastasis, are potent inhibitors of the downstream enzyme of the mevalonate pathway farnesyl pyrophosphate synthase, thereby leading to the intracellular accumulation of upstream metabolites as IPP and in consequence to Vγ9Vδ2 T cell activation (13, 14). On the contrary, statins block the upstream enzyme hydroxymethylglutaryl-CoA reductase and inhibits IPP production, inhibiting Vγ9Vδ2 T cell activation (15).

Vγ9Vδ2 T cells express numerous molecules potentially associated with different stages of differentiation, migration, and functions. Vγ9Vδ2 T cells include “naive” and “central memory” phenotypes (T_Naive_, CD45RA^+^CD27^+^; T_CM_, CD45RA^−^CD27^+^) that home to secondary lymphoid organs, but lack immediate effector function, and “effector/memory” (T_EM_, CD45RA^−^CD27^−^) and “terminally differentiated” (T_EMRA_, CD45RA^+^CD27^−^) phenotypes that home to sites of inflammation and display immediate effector function (16).

While T_Naive_ and T_CM_ cells readily respond to PAgs stimulation, T cells with effector memory (T_EM_) and terminally differentiated effector memory (T_EMRA_) expand in response to homeostatic cytokines as IL-15 (17) and differentiate in the presence of polarizing cytokines (18). On activation, Vγ9Vδ2 cells can be skewed toward distinct effector functions depending on polarizing cytokines, in analogy to CD4 helper T cells. Typically, human Vγ9Vδ2 T cells default toward type 1 cytokine production (γδ1), but under appropriate culture conditions they divert from the typical γδ1 phenotype and polarize to γδ2 (19, 20), γδ17 (21–23), γδ_FH_ (24, 25), and γδreg cells (26). Such a broad plasticity emphasizes the capacity of Vγ9Vδ2 T cells to influence the nature of immune response to different challenges.

## γδ T Cells for Tumor Immunotherapy

The major goal of tumor immunotherapy is the induction of adaptive responses of B cells and MHC-restricted αβ T cells, particularly CD8 cytotoxic T cells. Nonetheless, despite major advances in this area, durable responses are rare and immunotherapy is not yet an established modality to treat tumors. Furthermore, tumors frequently develop strategies to escape immune responses (27, 28). In contrast to αβ T cells, γδ T cells have unique features (see Table 1), which makes them good candidates for effective tumor immunotherapy. For instance, they lack MHC restriction, and do not require co-stimulation. Therefore, common tumor antigens without MHC restriction provide broader applicability of γδ T cells across a wide range of tumors and patients with diverse MHC alleles. Moreover, γδ T cells display potent cytotoxic and anti-tumor activity *in vitro* (29–33) and in xenograft models *in vivo* (34, 35). Cytotoxicity of γδ T cells against tumor cells is associated with increased production of PAgs (36), which is, at least, partly due to the increased expression of hydroxymethylglutaryl-CoA reductase, the rate limiting enzyme of the mevalonate pathway (36). Moreover, intracellular levels of IPP can be manipulated by aminobisphosphonates (13–15, 37–39), thereby leading to the intracellular accumulation of IPP and in consequence to activation of Vγ9Vδ2 T cells (36). As above discussed, in addition to the binding of the antigenic molecules to the reactive TCR, Vγ9Vδ2 T cells express NK cell activating receptors such as NKG2D, which recognizes target cells expressing MICA, MICB, and ULBPs (3, 4, 40, 41). These interactions may prove crucial in Vγ9Vδ2 T cell recognition and killing of tumors of hematopoietic origin. In fact, the expression levels of ULBP1 determine lymphoma susceptibility to Vγ9Vδ2 T cell-mediated cytolysis, highlighting a thus far unique physiologic relevance for tumor recognition by Vγ9Vδ2 T cells (42, 43).

**Table 1:** **Advantages of using γδ T cells for tumor immunotherapy**.

The frequency of γδ T cells is very high (1–5/10^2^), compared to that of Ag-specific αβ T cells (1/10^5^–10^6^)
γδ T cells can recognize and lyse a broad range of tumor cells and there is no need to target tumor-specific Ags
γδ T cells lack MHC restriction in antigen recognition
γδ T cell activation does not require co-stimulatory signals (e.g., CD28)
mAb can be used *in vivo* to enhance γδ T cell cytotoxicity (ADCC)
γδ T cells seem to be devoid of GVH activity
FDA-approved drugs (Zoledronate, IL-2) available for γδ T cell expansion *in vivo*
Large scale *ex vivo* expansion of γδ T cells and clinical grade sets for purification

After recognizing target cells via the TCR, or NKG2D, or both, Vγ9Vδ2 T cells preferentially use the perforin/granzyme (44) and/or TRAIL (45) pathways, as well as the Fas/FasL killing signal (46), for cytotoxicity against target cells like tumor cells. In addition, activated Vγ9Vδ2 T cells secrete IFN-γ and TNF-α, which have cytotoxic activity against tumor cells directly and indirectly *via* stimulating macrophages and DCs (47–49).

Overall, the potent anti-tumor activity of Vγ9Vδ2 T cells and their wide reactivity to several tumor cell types has led to the exploration of their therapeutic potential. Two strategies have been developed to apply the anti-tumor activities of Vγ9Vδ2 T cells to cancer immunotherapy: (1) *in vivo* administration of compounds that activate Vγ9Vδ2 T cells and (2) adoptive transfer of *ex vivo*-expanded Vγ9Vδ2 T cells. Several small-sized clinical trials have tested the efficacy of any of these two strategies in patients with various tumor types and a recent meta-analysis based on data from 13 clinical trials including a total of 204 patients has demonstrated that Vγ9Vδ2 T cell-based immunotherapy improves overall survival and, in view of its low toxicity grade (50), provides a proof of principle for its utilization as adjuvant to conventional therapies.

## Tumor-Infiltrating γδ T Cells and Their Correlation to Cancer Outcome

Tumor-infiltrating leukocytes are an heterogeneous population of immune cells that have been found in a wide variety of solid tumors (51) and the extent of leukocyte infiltration has been often associated with improved prognosis (52). However, there is a limited number of studies regarding the contribution of individual leukocyte subsets to survival. Tumor-infiltrating leukocytes include cells of the myeloid lineage (granulocytes, macrophages, and myeloid-derived suppressor cells) and several different lymphocyte subsets (T, B, and NK), each with different impact on tumor progression. Results of mouse tumor models and human cohort studies have suggested that any individual leukocyte population may correlate with poor or better prognostic factors, such as tumor stage/grade, presence of metastasis, and disease-free/overall survival. In general, infiltration by myeloid cells has been associated with tumor progression, while the presence of abundant T cells (particularly of the CD8 subset) is associated with tumor regression and improved prognosis. However, the limits of the immunohistochemical techniques largely used in retrospective clinical studies, have so far prevented a detailed descriptions of different tumor-infiltrating leukocyte populations as well as evaluation of their functional properties in the tumor microenvironment. For instance, tumor-infiltrating T lymphocytes may be endowed with regulatory function and hence promote tumor progression.

Several studies have shown that γδ T cells are present among tumor-infiltrating lymphocytes (TILs) from patients affected by different types of cancer, but their clinical relevance remains still obscure because of conflicting results obtained.

In detail, there have been five relatively recent large studies, which have correlated tumor-infiltrating γδ T cells with several different clinical features:

Bialasiewicz et al. (53) evaluated by immunohistochemical analysis TILs in 113 specimens from patients with necrotizing choroidal melanoma. They detected TILs in 76% of samples and γδ T cells, mainly of the Vδ1 subset were present in 52% of samples. Most notably, the presence of γδ T cells in tumors positively correlated with patient’s survival, indicating that tumor-infiltrating γδ T cells are a prognostically favorable factor.

Inman et al. (54) assessed by immunohistochemical analysis total γδ T cells in 248 renal cancer specimens and correlated these values with clinicopathologic prognostic factors and cancer outcome. They found that percentages of intratumoral γδ T cells were usually very low (<1% of the CD3^+^ population) in nearly all tested tumor specimens and did not correlate with any examined prognostic factor or even with survival. Authors concluded that the role of γδ T cells in renal cancer is questionable.

Ma et al. (55) examined by immunohistochemistry total γδ T cells infiltrating breast cancer in specimens of 46 patients. γδ T cells were detected in nearly all cancer patients (93%), but only in 3% of normal breast specimen. Authors did not quantify the percentages of intratumoral γδ T cells, but when an arbitrary cut-off of nine γδ T cells per high magnification microscopic field was used to define TIL-high (>9) and TIL-low (<9) groups, authors found that γδ T cell numbers were positively correlated with advanced tumor stages, HER2 expression status, and high lymph node metastasis, but inversely correlated with relapse-free survival and overall survival of patients. Multivariate and univariate analysis of tumor-infiltrating γδ T cells and other prognostic factors further suggested that intratumoral γδ T cells represented the most significant independent prognostic factor for assessing severity of breast cancer compared with the other known factors. Authors concluded that tumor-infiltrating γδ T cells play a crucial role in breast cancer progression and pathogenesis and may serve as a valuable and independent prognostic biomarker for human breast cancer.

Cordova et al. (56) studied the representation of tumor-infiltrating γδ T cells from 74 patients with primary melanoma. γδ T cells were the major subset among CD3^+^ T lymphocytes and comprised equal percentages of Vδ1 and Vδ2 T_EM_ or T_EMRA_ phenotypes. In this study, the presence of γδ T cells, and in particular the Vδ2 subset, among TILs significantly correlated with early stage melanoma, while percentages of infiltrating Vδ1 T cells did not correlate with any examined prognostic factor of melanoma.

Finally, Wu et al. (57) demonstrated that γδ T cells (γδ17) are the major source of IL-17 in human colon cancer, with the majority (80%) of the IL-17^+^ γδ T cells expressing Vδ1 and 20% expressing Vδ2. Importantly, analyzing 117 colon cancer samples, authors found that γδ17 cell infiltration positively correlated with tumor stages and other clinicopathological factors (tumor size, tumor invasion, lymphatic and vascular invasion, lymph node metastasis, and serum CEA levels), indicating that tumor-infiltrating γδ17 T cells are associated with tumor invasiveness and progression and may thus represent a prognostic factor in human colon cancer.

## Tumor-Infiltrating γδ T Cells: What are They and What Do They Do ?

The above discussed findings that tumor-infiltrating γδ T cells correlate with tumor remission, or with tumor progression or even fail to correlate with any prognostic feature strongly suggest that γδ T cells in the tumor microenvironment may play substantially different functions; hence positive or negative correlation with prognosis may depend on the specific γδ T cell subset/function recruited at the tumor site. Furthermore, the net biologic effects of γδ T cells may depend on the tumor type and the tumor site, perhaps reflecting microenvironmental differences: for instance TGF-β, which is abundantly secreted at the tumor site by tumor-infiltrating macrophages or by tumor cells themselves, may favor the differentiation of γδ cells with Treg-like properties, which in turn inhibit anti-tumor immune responses.

Initial studies on the functional properties of tumor-infiltrating γδ T cells were performed using polyclonal γδ T cell lines generated *in vitro* upon long term culture with mitogen/antigen and IL-2: this approach was mainly due to the very low number of γδ T cells recovered from tumor specimen and to the lack of suitable techniques, which allowed precise detection of functional markers. These studies have unequivocally demonstrated that *ex vivo*-expanded γδ T cell lines and clones from renal, breast, lung, ovary, colon, and pancreatic cancer efficiently kill stabilized tumor cell lines and freshly isolated tumor cells and generally Vδ1 T cell lines had the higher cytotoxic activity compared to Vδ2 T cell lines (58–63). Accordingly, Cordova et al. (56) confirmed these results using polyclonal γδ T cell lines derived from melanoma; both Vδ1 and Vδ2 T cell lines produced equal amounts of TNF-α and IFN-γ, but while the majority (75%) of Vδ1 T cell lines exerted potent cytotoxic activity against melanoma cell line *in vitro*, only 25% of the Vδ2 T cell lines showed appreciable lytic activity. Therefore, based on their cytolytic activity and production of cytokines with proven anti-tumor effect, tumor-infiltrating γδ T cells have been long regarded as important players of the anti-tumor immune response. However, both the failure to consistently detect a positive correlation between the presence of γδ T cells in the tumor microenvironment and the patient’s prognosis, as well as the improvement of immunological techniques to detect functional signatures even in very small tissue samples have subverted the concept that γδ T cells are simply an important component of resistance to cancer and suggested that their function may be extremely pleiotropic and including either effector or suppressive potential.

In 2007, Peng and colleagues (64) unexpectedly identified a Vδ1^+^ population (which comprised over 95% of the total γδ T cells population) among breast cancer-infiltrating lymphocytes capable to suppress immune responses. In particular, Vδ1 cells inhibited CD4 and CD8 T cell activation and impaired DC maturation and function. Although the mechanisms responsible for the regulatory activity of tumor-infiltrating Vδ1 cells was not investigated in that paper, it seems to involve TLR8 signaling pathway, as suppression was reversed by TLR8 ligands. Later on, the same group reported (65) that breast cancer-infiltrating Vδ1 cells induced both T cell and DC senescence and the senescent T cells and DCs in turn became regulatory cells, thus determining amplification of the immunosuppressive process. Interestingly, and surprisingly, accumulation of regulatory Vδ1 cells in the context of breast cancer (where they account for approximately 30% of the total lymphocyte population) is not due to proliferation of resident Vδ1 cells but to their recruitment mediated by IP-10 secreted by breast cancer cells.

In addition to the above quoted studies on tumor-infiltrating human regulatory γδ T cells, four recent reports, three in mice and one in humans, have shed light on the regulatory role played by IL-17-producing γδ T cells (γδ17) and have also defined the underlying mechanisms.

Using a transplantable tumor mouse model, Wakita et al. (66) observed that γδ T cells accounted for 25% of all TILs and selectively produced IL-17 but not IFN-γ. Importantly, absence of IL-17 caused inhibition of tumor growth, which correlated with a reduced number of blood vessels within the tumor and reduced expression levels of VEGF and Ang-2 in tumor cells. This indicates that tumor-infiltrating γδ17 T cells promote angiogenesis, and thus tumor growth.

A similar detrimental effect of IL-17 has been reported by Ma and colleagues (67) in an hepatocellular carcinoma mouse model. Similarly to the findings of Wakita et al., γδ T cells were the major source of IL-17 amongst lymphocytes infiltrating hepatocellular carcinoma. In this model, absence of IL-17 reduced tumor growth, while its administration promoted the growth of hepatocellular carcinoma. However, the mechanism responsible for the anti-tumor activity of IL-17 was different from that reported by Wakita and involved a reciprocal activatory interaction between the γδ17 cells and MDSC, which was mediated by cancer cells: in detail, γδ T cell-derived IL-17 induced CXCL5 production by tumor cells, which in turn recruited MDSC to the tumor sites *via* CXCL5/CXCR2-interaction. Once at the tumor site, IL-17 induced production of IL-1β and IL-23 in MDSC, which amplify differentiation of γδ17 cells. This positive feedback between γδ17 cells and MDSC sustains immunosuppression and promotes tumor growth.

The third mouse study by Silva Santos and colleagues (68) used a transplantable peritoneal/ovarian cancer, and confirmed the crucial role of γδ17 in promoting cancer growth. γδ17 accumulated in the peritoneal cavity and were the main source of IL-17 also in this model. γδ17 caused the recruitment at the tumor site of an unconventional population of small macrophages that expressed IL-17 receptor and a number of pro-tumor and pro-angiogenic molecules amongst which VEGF and TGF-β, which promoted cancer cell proliferation and tumor growth.

The fourth study on the participation of γδ17 cells in cancer was performed by Wu et al. (57) in human colorectal cancer. In that study, tumor-infiltrating γδ T cell was the main source of IL-17 and 80% of the γδ17 cells expressed Vδ1. Of note however, γδ17 constituted approximately 25% of all tumor-infiltrating Vδ1 cells and co-produced TNF-α, IL-8, and GM-CSF. All these cytokines, in different combinations, caused recruitment (IL-8 and GM-CSF) and survival, activation, and proliferation (TNF-α, IL-8, and IL-17) of MDSC that in turn mediate immunosuppression and promote tumor growth.

Altogether these results clearly demonstrate that γδ17 cells are key mediators of tumor-associated immunosuppression thereby influencing tumor progression.

## Hypothesis: Tumor Microenvironment as the Critical Determinant of Tumor-Infiltrating γδ T Cell Fate

The conditions under which γδ T cells can contribute to tumor control versus immune suppression need to be defined. There are several theoretical possibilities to answer the fundamental question of the molecular mechanisms that explain these two γδ T cell phenotypes.

First, it is possible that genetic differences in tumor cells influence the host response, through the involvement of different pathways that are mutated or activated in a heterogeneous fashion and that regulate the expression of immune system regulatory genes. For instance, tumor cells with STAT3 activation show impaired production of chemokines and cytokines, but increased production of immunosuppressive factors and thus escape immune recognition (69).

Second, it is possible that polymorphism of regulatory genes might influence lymphocyte activation at the tumor site. For instance, IRF5 polymorphism is associated with clinical response to adoptively transferred TILs in melanoma patients (70).

Third, it is likely that exposure to certain pathogens or even the intestinal microbiome could change the frequency, phenotype, and functions of TILs. For instance, Wu et al. (57) showed that in colon cancer patients, destruction of the epithelial barrier caused by tumor development results in tumor invasion by commensal bacteria (*E. coli*) and release of bacterial product, which promote IL-23 production by DCs and γδ17 cell polarization *in situ*.

**Figure 1:**
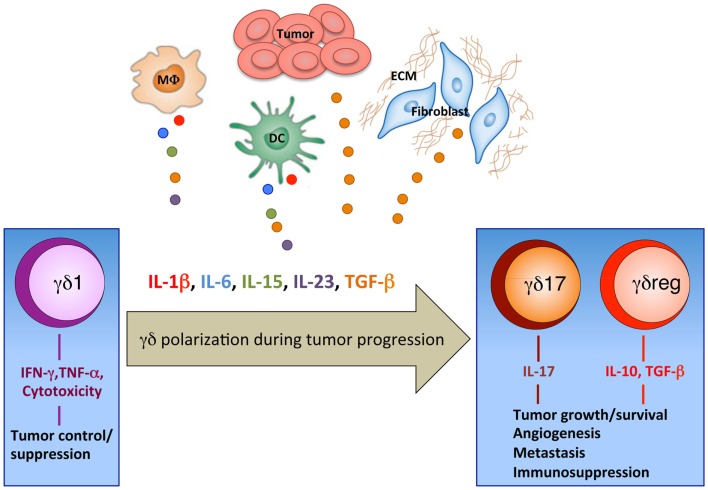
**Polarization of γδ T cells in the tumor microenvironment**. Our working hypothesis for the recruitment of γδ T cells with different phenotypes and functions into the tumor site. At early stages of tumor development tumor cells produce chemokines, which recruit γδ T cells of the γδ1 type equipped with anti-tumor activities (IFN-γ and TNF-α production and cytotoxic potential). It is speculated that during progression, tumors have denser stroma (ECM, extracellular matrix) and alternative DCs, myeloid, or macrophage (MΦ) populations, which produce cytokines that accumulate in the tumor microenvironment and cause polarization of γδ cells from γδ1 to γδ17 and γδreg.

Fourth, it is likely (and this is the possibility we favor) that tumor microenvironment plays a key role. By definition, tumor microenvironment is a complex network of different cell types, soluble factors, signaling molecules, and extracellular matrix components, which orchestrate the fate of tumor progression (71). In fact, in addition to the tumor cells and to the several lymphoid and myeloid cell types that infiltrate tumors, classical cellular components of the solid tumor stromal microenvironment also influence the host immune response. The tumor stroma consists of fibroblasts, macrophages, and vascular endothelial cells, with variable amounts of extracellular matrix, all of which contribute not only as a support structure for tumor growth, but can also impair host immune responses and likely contribute to the quality of immune cell infiltration (71). We hypothesize (Figure 1) that, at early stages of tumor development γδ T cells of the γδ1 type producing cytokines with proven anti-tumor activity (IFN-γ and TNF-α) and equipped with cytotoxic potential either expand locally (Vδ1) or are recruited at the tumor site from peripheral blood (Vδ2) and may exert anti-tumor activity; however, with tumor progression, factors produced in the microenvironment cause polarization of γδ cells from γδ1 to γδ17 and γδreg, which instead promote tumor progression. A plethora of cell types present in the tumor microenvironment may actually provide the source of such γδ cells polarizing factors.

For instance, colon cancer stem cells and tumor-associated macrophages and fibroblasts produce huge amounts of TGF-β (72) which, in combination with other cytokines present in the microenvironment, contributes to polarization of γδ T cells to γδ17 and γδreg.

Macrophages, DCs, and other myeloid cells, which are typically found in the solid tumor microenvironment, produce IL-15, which in combination with TGF-β determines γδreg polarization (26), and IL-1β, IL-6, IL-23, and TGF-β, which in different combination promote γδ17 Polarization (22).

Finally, it is also possible that those γδ T cells within TILs equipped with anti-tumor activities die after antigenic activation and the frequencies of γδreg, which would be less tumor reactive or resistant to cell death then increase. Moreover, nitric oxide, which is largely produced by MDSC in the tumor microenvironment may contribute to apoptosis of γδ T cells induced by antigen activation (73).

**Figure 2:**
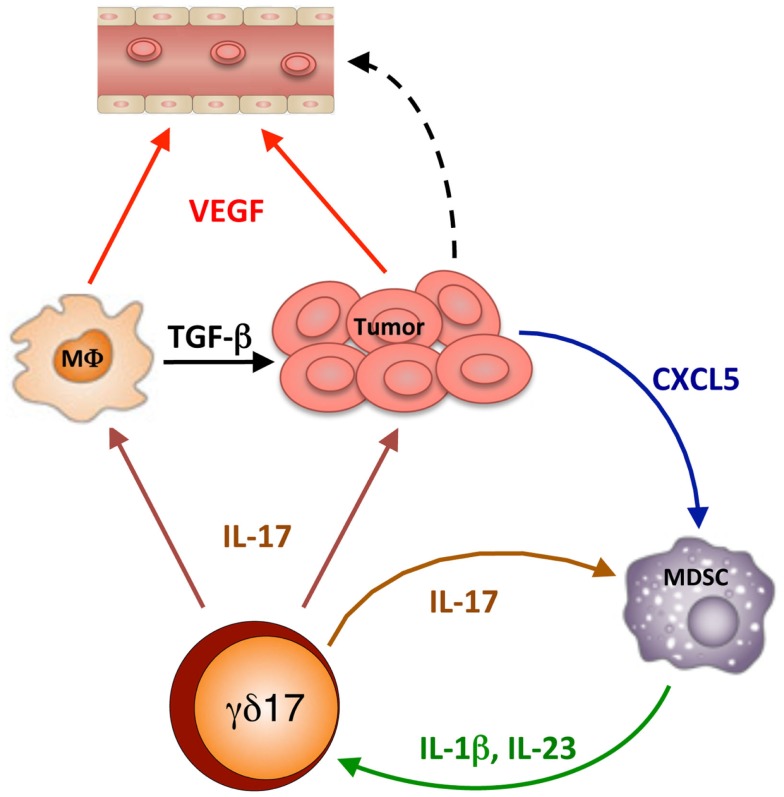
**IL-17-dependent cellular interactions into the tumor microenvironment**. γδ17 release IL-17, which mediates several effects: it promotes VEGF production by cancer cells and macrophages, thus causing tumor vascularization and favoring metastasis; it promotes TGF-β production by macrophages (MΦ) thus causing tumor growth; it promotes CXCL5 production by tumor cells which, in turn, recruited MDSC. Then a mutual stimulatory circuit is established between MDSC and γδ17, which involves IL-17, IL-1β, and IL-23, which leads to further differentiation and activation of γδ17 T cells and amplification of the immunosuppressive process.

Once activated, γδreg and γδ17 amplify the immunoregulatory process in different ways (Figure 2): IL-17 promotes VEGF production by cancer cells and macrophages, and CXCL5 production by tumor cells which, in turn, recruited MDSC. An activatory cross-talk is then established at the tumor site between MDSC and γδ17, by which IL-17 induces IL-1β and IL-23 production by MDSC and these cytokines promote further differentiation and activation of γδ17 T cells. Finally, γδreg produce IL-10 and TGF-β, which act on several cellular targets to promote immunosuppression at the tumor site and favor tumor progression.

Several recent discoveries have been made toward understanding the biological effects of cytokines, particularly TGF-β, produced in the tumor microenvironment that can polarize many arms of the immune system. Thus, and similarly to what we propose to occur for γδ cells, cytokines present in the tumor microenvironment induce DCs to acquire a tolerogenic phenotype, convert N1 neutrophils to a N2 phenotype and promote the recruitment of M2 over M1 macrophages. Within the tumor microenvironment, cytokines also inhibit Th1 and CD8 CTL functions and probably promote a shift toward Th2, Th17, and Tc17 differentiation and convert CD4 effector T cells to induced Treg (iTReg) cells. For a systematic review on the polarization of immune cells in the tumor microenvironment see in Ref. (74).

## Conclusion and Perspectives

The mutual and interdependent interaction between tumor and its microenvironment is a crucial topic in cancer research and therapy, as recently demonstrated by the finding that targeting stromal factors could improve efficacies of current therapeutics and prevent metastasis. For instance, combinatorial therapy with an agonistic mAb against CD40 and standard gemcitabine chemotherapy, proved unexpectedly efficient in pancreatic cancer (75). In this system, the anti-CD40 mAb caused massive recruitment of macrophages at the tumor site, which caused severe disruption of the tumor stroma thus allowing increased concentrations of gemcitabine to accumulate to the tumor site (75). Similarly, the identification of defined immunosuppressive pathways in the tumor microenvironment has pointed toward therapeutic targets that are amenable to clinical intervention: these include, for instance, mAbs to PD-1 or PD-L, CTLA-4, CD25, and small-molecule inhibitors that block IDO enzymatic activity [reviewed in Ref. (76)]. These novel strategies must be kept in mind when designing γδ T cell-based therapy. Moreover radiation therapy, low dose traditional chemotherapeutic drugs and aminobisphosphonates not only sensitize tumor cells to immune recognition and killing, but also modulate the tumor microenvironment and contribute to the therapeutic effect (77–80): for instance, zoledronic acid at clinically achievable doses repolarizes tumor-associated macrophages to a M1 phenotype and reduces the number of MDSC (81).

γδ T cell-based immunotherapy is emerging to be a powerful treatment option for patients with different types of tumors. It includes the *in vivo* activation of γδ cells or the adoptive transfer of *ex vivo*-expanded γδ cells. Although we have no evidence of the fate of the activated γδ cells and the long term effects of the γδ cell-based therapies in preclinical and clinical studies, it is reasonable to predict that the tumor microenvironment plays an indispensable role in limiting the effectiveness of γδ T cell-based immunotherapies. Additionally, the local expansion of adoptively transferred γδ T cells has to be increased to achieve higher T cell numbers at the tumor site.

Therefore, combination regimens consisting of γδ T cell-based therapies and strategies aimed to circumvent the negative impact of the tumor microenvironment onto γδ T cells and induce γδ T cell repolarization, may prove efficacious and achieve clinical benefit in patients with different types of tumor.

## Conflict of Interest Statement

The authors declare that the research was conducted in the absence of any commercial or financial relationships that could be construed as a potential conflict of interest.
